# Preclinical Efficacy and Toxicology Evaluation of RAC1 Inhibitor 1A-116 in Human Glioblastoma Models

**DOI:** 10.3390/cancers14194810

**Published:** 2022-09-30

**Authors:** Georgina A. Cardama, Julian Maggio, Lucas Valdez Capuccino, Nazareno Gonzalez, Valentina Matiller, Hugo H. Ortega, German R. Perez, Ignacio A. Demarco, Eduardo Spitzer, Daniel E. Gomez, Pablo Lorenzano Menna, Daniel F. Alonso

**Affiliations:** 1 Centro de Oncología Molecular y Traslacional (COMTra), Departamento de Ciencia y Tecnología, Universidad Nacional de Quilmes, Bernal B1876BXD, Argentina; 2 Consejo Nacional de Investigaciones Científicas y Técnicas (CONICET), Godoy Cruz 2290, Buenos Aires C1425FQB, Argentina; 3 Instituto de Investigaciones Biomédicas (INBIOMED, UBA-CONICET), Facultad de Medicina, Universidad de Buenos Aires, Buenos Aires C1121ABG, Argentina; 4 Centro de Medicina Comparada, Instituto de Ciencias Veterinarias del Litoral (ICiVet-Litoral), Universidad Nacional del Litoral (UNL)/Consejo Nacional de Investigaciones Científicas y Técnicas (CONICET), Santa Fe S3080HOF, Argentina; 5 Facultad de Ciencias Bioquímicas y Farmacia, Universidad Nacional de Rosario, Santa Fe S2002LRK, Argentina; 6 MabXience S.A.U, Buenos Aires B1619, Argentina; 7 Laboratorio Elea/Phoenix, Los Polvorines B1613AUE, Argentina; 8 Laboratorio de Farmacología Molecular, Universidad Nacional de Quilmes, Bernal B1876BXD, Argentina

**Keywords:** Rac1 inhibitor, glioma, 1A-116

## Abstract

**Simple Summary:**

Malignant gliomas are the most common primary central nervous system tumors in adults. Currently, this disease is associated with poor prognosis and is virtually incurable. There is a need to find novel targets and treatments to improve patient survival. This study shows the preclinical evaluation of 1A-116, a Rac1 inhibitor that showed in vitro antitumor activity on glioma cells. We also evaluated 1A-116 in vivo, showing a favorable toxicological profile and antitumor efficacy in an intracranial mouse tumor model. Altogether, our study provides important evidence of 1A-116 as a signal transduction-based precision therapy for glioma and also increases the evidence of Rac1 as a key molecular target in cancer.

**Abstract:**

Malignant gliomas are the most common primary central nervous system tumor in adults. Despite current therapeutics, these tumors are associated with poor prognosis and a median survival of 16 to 19 months. This highlights the need for innovative treatments for this incurable disease. Rac1 has long been associated with tumor progression and plays a key role in glioma’s infiltrative and invasive nature. The aim of this study is to evaluate the 1A-116 molecule, a Rac1 inhibitor, as targeted therapy for this aggressive disease. We found that targeting Rac1 inhibits cell proliferation and cell cycle progression using different in vitro human glioblastoma models. Additionally, we evaluated 1A-116 in vivo, showing a favorable toxicological profile. Using in silico tools, 1A-116 is also predicted to penetrate the blood–brain barrier and present a favorable metabolic fate. In line with these results, 1A-116 i.p daily treatment resulted in a dose-dependent antitumor effect in an orthotopic IDH-wt glioma model. Altogether, our study provides a strong potential for clinical translation of 1A-116 as a signal transduction-based precision therapy for glioma and also increases the evidence of Rac1 as a key molecular target.

## 1. Introduction

Malignant gliomas are the most common type of primary malignant brain tumors. The current standard of care combines maximal surgical resection, followed by radiotherapy with concomitant and adjuvant chemotherapy based on the alkylating agent temozolomide (TMZ) [1]. Despite this multimodal therapy, these tumors are associated with poor prognosis and a median survival of 16 to 19 months. Glioblastomas (GBM) are highly aggressive with heterogeneous molecular features and diverse phenotypes that translate into a remarkable disease complexity, which includes genetic anomalies, epigenetic dysregulation, cellular plasticity, immunosuppression, and metabolic adaptability. Histologically, gliomas are characterized by high cellularity, vascular proliferation, and necrosis. Glioma cells are invasive and infiltrative, and this key feature has been associated with tumor recurrence and resistance to treatment, highlighting the limited therapeutic options available and the need for innovative treatments for this incurable disease [2].

Rac1 GTPase is one of the most extensively studied members of the Rho GTPase family of proteins [3]. Rac1 is a molecular switch with a key role in the regulation of actin dynamics, cell proliferation, migration, invasion, and apoptosis. Moreover, it has been associated with tumor progression and resistance mechanisms in various tumor types, including malignant gliomas [4]. Interestingly, Rac1 and its downstream effector PAK1 have been reported to be involved in the maintenance of glioma stem-like cells and tumorigenicity in human glioma [5]. These key roles in malignant progression highlight Rac1 as an interesting therapeutic target in glioma [6].

We have previously shown the development of 1A-116 Rac1 inhibitor using a rational design approach. 1A-116 was able to modulate the Rac1 signaling pathway and affect Rac1-driven cell activities such as cell proliferation, cell cycle progression, cell invasion, and apoptosis in different tumor models [7,8]. Importantly, 1A-116 has been shown to modulate Rac1-GTP levels by blocking Rac1 interaction with different GEFs, such as Vav, Tiam1, and DBL. 1A-116 activity depends on the presence of W56 residue in Rac1 structure, having no effect on the closely related Rho GTPase Cdc42. The 1A-116 antitumor effect was previously reported by us and others in several cancer types [7,8,9,10,11]. In human glioma cells, we have shown that Rac1 inhibition was able to promote apoptosis and inhibit invasion and the lack of Rac1 by siRNA reduced 1A-116 activity [12].

In this study, we show the preclinical development of a 1A-116 Rac1 inhibitor using relevant cell culture systems and animal models to further evaluate the translational potential of this compound, focusing on efficacy and toxicology. For this purpose, we first used a panel of GBM cell lines with diverse molecular features and also evaluated the effect of 1A-116 on 3D spheroid proliferation. The 1A-116 toxicology profile was also examined, and pharmacokinetic features of the molecule were predicted using in silico tools. These results and the dose-dependent efficacy shown in a mouse orthotropic intracranial IDH-wild type glioma model suggest that 1A-116 may be a useful novel therapeutic agent for glioma treatment, highlighting Rac1 as a valuable target in glioma.

## 2. Materials and Methods

### 2.1. Genomic Database Analysis

RNA-Seq expression data of Rac1 (Illumina HiSeq 2000 RNA sequencing platform) from 420 low-grade gliomas (LGG, IDH1 mutated (mIDH1) samples and 154 GBM samples were obtained from The Cancer Genome Atlas (TCGA) and analyzed using the UCSC Xena browser [13]. This database provides quantitative gene expression information and a compelling list of patients’ characteristics, including their clinical parameters and survival rates. Patients were divided into two groups with low or high Rac1 expression using the median expression level of all cases as the cut-off point. GraphPad Prism version 8 software was used for survival analyses. Kaplan–Meier curves were employed to estimate the overall survival distribution between groups.

### 2.2. Cell Lines

Human glioma cell lines LN229, U-87 MG, U252, U373, LN18, A172, and T98G were obtained by our collaborators from ATCC. Importantly, Rac1 levels have already been characterized, confirming the target presence in all cell lines [5,14,15,16,17]. Cells were grown in Dulbecco’s Modified Eagle’s Medium (DMEM) supplemented with 10% heat-inactivated fetal bovine serum, 2 mM glutamine, and 80 μg/mL gentamicin at 37 °C in a 5% CO2 atmosphere. Cell subculture was routinely carried out twice a week by trypsinization using standard procedures.

### 2.3. Cell Proliferation Assay

First, 3.5 × 10^4^ cells were plated in 96-well plates and treated with different concentrations of 1A-116 (5 µM, 10 µM, 25 µM, 50 µM, 100 µM). After 72 h, cells were fixed with methanol and stained with crystal violet 0.5%. For measurement, crystal violet was resuspended in Methanol-Acetic acid (3:1), and absorbance was measured at 595 nm. The concentration producing 50% inhibition (IC50) was determined by the non-linear regression function of GraphPad Prism6.

### 2.4. 3D Spheroid Cell Culture Growth

For the spheroid induction, we used the Hanging Drop method. Briefly, 20 µL of complete medium with 5000 LN229 cells or 5000 U87-MG cells were seeded on the lid of a culture dish and allowed to form a spheroid. After 72 h, the resulting cellular structures were transferred to a 96-well plate that had previously been coated with agar 1.5% to avoid attachment of the cells to the plastic. After approximately 7 days after the transfer, spheroids showed proper sphericity [18] and a minimum diameter of 200 µm. Once established, they were randomly separated into groups and treated. This consisted of replacing 2/3 of the media with fresh complete media with different concentrations of 1A-116. The treatment was carried out twice a week for 17 days. Twice a week, the spheroids were photographed to have an individual follow-up of each one and to relativize spheroid volume during the experiment to the initial day of treatment. For the analysis of the spheroids, we used the open-source software AnaSP and ReViSP [18]. This software allows us to calculate the volume of each spheroid from the photographs taken.

### 2.5. Cell Cycle Analysis

For cell cycle analysis by flow cytometry, cells were washed and incubated in serum-free DMEM for synchronization for 48 h. Cells were then treated for 24 h with different concentrations of 1A-116 in D-MEM supplemented with 10% fetal bovine serum and collected by trypsinization. Cells were fixed in 70% methanol in PBS and stained with propidium iodide (1 mg/mL) (Thermo Fisher Scientific, Waltham, MA, USA). Cell cycle progression was analyzed in a BD FACSCalibur™ (BD Biosciences, San Jose, CA, USA) flow cytometer. Before recording 10,000 events, the verification of the doublet discrimination function of the flow cytometer was performed with a DNA QC Particles kit (BD Biosciences).

### 2.6. In Silico Prediction Tools: 1A-116 Pharmacokinetics

We focused on gastrointestinal absorption, blood–brain barrier penetration, P-gp substrate prediction, and molecule metabolism using cheminformatics. First, we used the SwissADME tool [19] to predict the first three parameters. Particularly, the BOILED-Egg model was used since it delivers a rapid, intuitive, easily reproducible yet statistically robust method to predict the passive gastrointestinal absorption and brain access of small molecules [20]. Additionally, we used the Biozyne software package to evaluate whether 1A-116 is predicted to be a P-glycoprotein (P-gp, MDR1) substrate. This protein is a promiscuous drug efflux pump of substantial pharmacological importance, especially in cancer therapeutics affecting the efficacy of antitumor agents [21]. Finally, XenoSite Web—P450 Metabolism [22] was used to predict the potential sites of metabolism (SOMs) in 1A-116. It uses a machine learning model to predict SOMs in small molecules and has proven to have good accuracy. This approach predicts SOMs for several P450 enzymes: CYP 1A2, 2A6, 2B6, 2C8, 2C9, 2C19, 2D6, 2E1, 3A4, and HLM. In all cases, the molecular structure of 1A-116 was uploaded in SMILES format to carry out the predictions.

### 2.7. Genotyping LN229 Cell Line: Clinically Relevant Genes

To establish the translational significance of in vivo efficacy with the LN229 model, clinically relevant genes were analyzed using MLPA (multiplex ligation-dependent probe amplification) described in Ruiz et al. [23]. The LN229 cell line was compared to normal neuronal tissue. Mutations in isocitrate dehydrogenase (IDH1 and IDH2), the methylation status of MGMT promoter, and clinically relevant amplifications and deletions (PDGFRA, EGFR, EGFRvIII presence, CDKN2A, PTEN, CDK4, MIR26A2, MDM2, NFKBIA, TP53) were determined for LN229 glioblastoma human cells.

### 2.8. Acute Toxicology and Non-Clinical Safety Studies

All the procedures were carried out according to the Guide for the Care and Use of Laboratory Animals (NRC, 2011) and with the approval of the Institutional Ethics and Security Committee (Protocol No. 332/16) of the School of Veterinary Science of the National University of Litoral, Santa Fe, Argentina. The Centre for Comparative Medicine is an entity compliant with GLP for conducting preclinical tests inspected by the Argentine Accreditation Organism (member of the OECD) and the certifications of local regulatory agencies such as the ANMAT (National Argentine Administration of Drugs, Food, and Medical Technology,) and the SENASA (National Argentine Service of Animal Sanitation and Food Quality).

Adult BALB/cCmedc mice (female, 6–7 weeks, 22.9 ± 0.8 g) were housed in individually ventilated cages (IVC) systems (Allentown Inc., Allentown, NJ, USA) and given food and water ad libitum. The temperature of the animal facility was 23 °C with a 12-h light/dark cycle.

To evaluate the safety of 1A-116, single injections of 31.2 and 68 mg/kg doses (n = 5 per dose) were administered to the treated animals via intraperitoneal (i.p) (10 mL/kg). Control animals (n = 5) received the same volume of saline solution. Animals were monitored daily for mortality and morbidity (clinical observations) and clinical pathology. On day 14, mice were sacrificed by anesthetic overdose, and a complete necropsy was carried out. Hematology and serum chemistry parameters were evaluated before carrying out the necropsy and before blood samples were taken from animals through a left ventricular puncture. Collected blood samples in EDTA tubes were analyzed using an automated hematology analyzer (Mindray BC-2800Vet, China). Haematologic parameters were analyzed, including red blood cells (RBCs), hematocrit (HCT), hemoglobin (HGB), white blood cell (WBC) with a differential count of neutrophils (NEU), eosinophils (EOS), basophils (BAS), lymphocyte (LYM), and monocyte (MNO).

Blood was also taken for serum chemistry in glass tubes, then placed for approximately 30 min at ambient temperature, and the serum was obtained by centrifugation at 3000 rpm for 10 min. Clinical chemistry was determined with validated micro methods for small volumes, with commercial kits (Wiener Labs, Argentina) and a microplate reader (SPECTROStar Nano, BMG Labtech, Germany). Alanine aminotransferase (ALT), aspartate aminotransferase (AST), gamma-glutamyltransferase (GGT), and amylase (AMY) were measured.

To determine the acute toxicity of 1A-116, an assay based on the Acute Toxicity—Up and down procedure was carried out in mice (n = 14) using the software AOT425StatPgm (Westat, EPA, USA) [24,25].

The test consisted of a single ordered dose progression in which animals were administrated, one at a time, at a minimum of 48-h intervals. For 1A-116, LD50 was estimated by developing a sequential test of 55 and 175 mg/kg via i.p. and 175, 500, and 2000 mg/kg via oral.

### 2.9. Glioblastoma Orthotopic Xenografts in Nude Mice

For efficacy studies, athymic female N:NIH(S) Fox1nu-/nu- mice aged 6–12 weeks (average weight of 20 g) were purchased from UNLP (Universidad Nacional de La Plata, Buenos Aires, Argentina) and housed at 5 mice per cage in our animal house facility at the National University of Quilmes. The animals were maintained in isolation cabinets with filtered air (HEPA 99.99%). Food and water were provided ad libitum, and the general health status of the animals was monitored daily. All animal protocols were approved by the ethics committee (CICUAL) of the National University of Quilmes: Protocol #011-15.

Using a stereotaxic device and its mouse and neonatal rat adaptor (Stoelting Co., Wood Dale, IL, USA), 2 × 10^5^ LN229 viable cells in a final volume of 2 uL were injected into the right striatum of the anesthetized mice with a 33 ga syringe (Hamilton) over the course of 3 min. The cells were injected 2.3 mm on the right, 0.1 mm posterior to the bregma, and 2 mm deep from the skull surface. After surgery, animals were allowed to recover, receiving antibiotics and analgesia. Then, 6 days after the operation, mice were randomly divided into groups, and treatment was started. The presence of the LN229 tumor was determined by histological examination (Figure S1, Supplemental Materials). Administration of 1A-116 was carried out in 200 µL doses with varying doses: 5 mg/kg/day, 10 mg/kg/day, 20 mg/kg/day. Following a 5-day per week schedule, doses were injected intraperitoneally (i.p.). The control group received 200 µL doses of the aqueous vehicle 5 times per week as well. Animals were euthanized by cervical dislocation when one of the following symptoms was reached: weight loss (>20%), poor physical condition, cachexia, improper moving, drowsiness, ataxia, bending hump posture, and other heavy neurological deficits symptoms.

## 3. Results

### 3.1. Rac1 Expression Correlates with Brain Tumor Grade and Poor Patient Outcome

**Figure 1:**
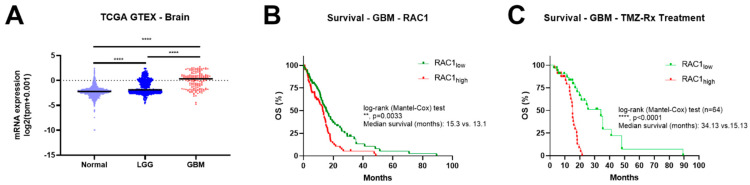
Rac1 levels in glioma patients from GTEX-TCGA LGG-GBM. (**A**) Transcriptomic data of Rac1 expression in normal tissue (GTEX) and in low-grade gliomas (LGG with IDH1 mutated) or GBM tumor biopsies. **, *p* < 0.001; ****, *p* < 0.0001. ANOVA, Tukey’s multiple comparisons test. (**B**) Overall survival (OS) curves of GBM patients stratified according to Rac1 levels (Rac1low vs. Rac1high). (**C**) Overall survival (OS) curves of GBM patients that received TMZ and/or radiotherapy (Rx) as treatment stratified according to Rac1 levels (Rac1low vs. Rac1high).

The expression of the Rac1 mRNA in low-grade gliomas (LGG, with IDH1 mutated) and GBM was analyzed using the GTEx and TCGA databases. In normal brain specimens, Rac1 mRNA expression is lower but is increased with increasing tumor grade and is significantly higher in GBM samples (Figure 1A). Additionally, clinical survival information was analyzed to determine the prognostic roles of Rac1 in GBM patients. As seen in Figure 1B, the group with low Rac1 expression had improved overall survival (OS) compared to high Rac1 expression (median survival 15.3 vs. 13.1 months). Importantly, high Rac1 expression resulted in worse OS in patients treated with temozolomide and/or radiotherapy (Figure 1C). These data suggest that high Rac1 expression correlates with poor patient outcomes and reveals Rac1 as a potential target in glioblastoma and that Rac1 could have a role in standard treatment response.

### 3.2. 1A-116 Rac1 Inhibitor Suppresses 2D and 3D Proliferation and Inhibits Cell Cycle Progression of Glioma Cells

**Figure 2:**
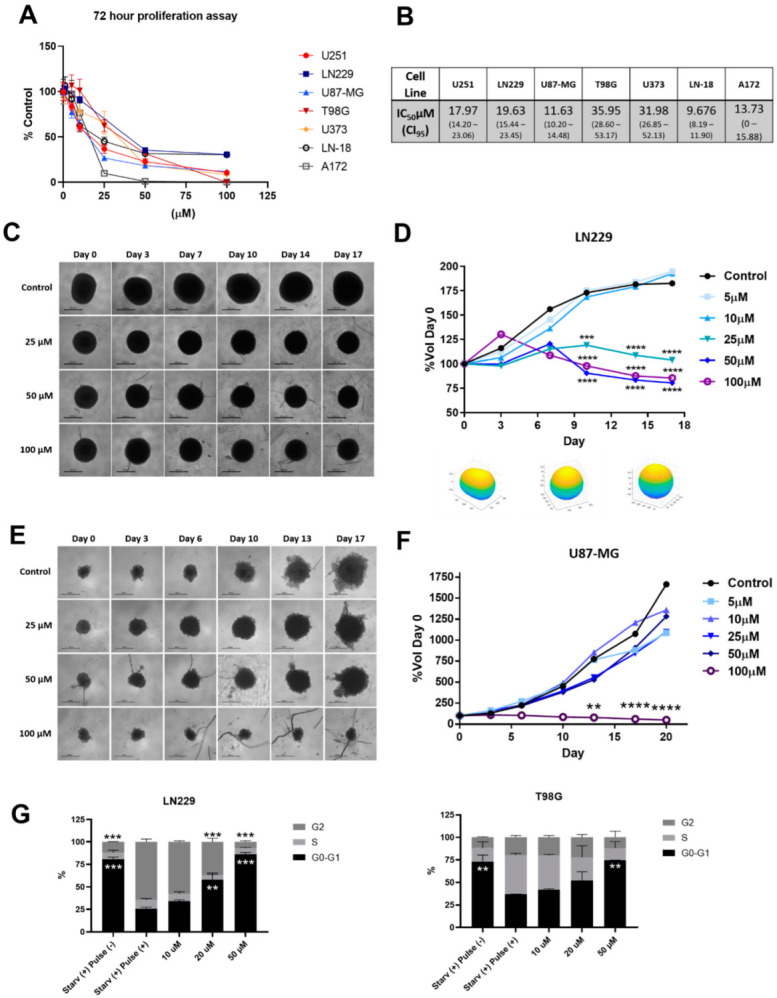
Rac1 inhibitor 1A-116 elicits in vitro antitumor activity on a panel of glioma cell lines. (**A**) Antiproliferative effect of 1A-116 on human glioma cell lines using a 2D setup, confirming 1A-116 effect on LN229 and U87MG cells. (**B**) Calculated IC50 values of 2D proliferation experiments. (**C**,**E**) Representative micrographs of LN229 cells (**C**) and U87MG (**E**) growing as spheroids in the presence of different 1A-116 concentrations. Scale bar: 100 μm. (**D**,**F**) Each spheroid was photographed over the course of the experiments, and volume was calculated. Each spheroid was relativized to day 0 volumes. Values are means ± SD (n = 6 spheroids/timepoint; a representative result of two indepen-ent experiments is shown). Two-way ANOVA, *** *p* < 0.001, **** *p* < 0.0001. (**G**) Cell cycle progression was analyzed by FACS to estimate the percentage of cells in the G1 phase, S phase, and G2/M phase. Columns, mean of a representative experiment (n = 3) of three independent experiments; bars, SD. ** *p* < 0.01. *** *p* < 0.001 determined by ANOVA cont. Tukey’s multiple comparison test.

We developed a 1A-116 Rac1 inhibitor using a rational design approach, aiming to inhibit Rac1 activation by its GEF activators [9]. Since Rac1 is an interesting molecular target in glioma [6], we assessed the effect of the 1A-116 Rac1 inhibitor on a panel of GBM cells. First, we evaluated and confirmed the effect of 1A-116 on 2D cell proliferation, and IC50 values were determined (Figure 2A,B). As seen in Figure 2A, 1A-116 presented a concentration-dependent antiproliferative effect in all evaluated cell lines.

We also investigated the effect of 1A-116 on 3D cultures of LN229 cells. Multicellular spheroids were established using the hanging drop method, and after reaching 200 μm diameter, spheroids were treated with different concentrations of 1A-116. After a 17-day treatment scheme, 1A-116 concentrations over 25 μM significantly abrogated spheroid growth (Figure 2C,D). This behavior was not seen in U87-MG cells, where only 100 μM 1A-116 was able to block growth completely (Figure 2E,F).

The proliferation arrest in 2D and 3D settings upon 1A-116 treatment may be accompanied by an onset of apoptotic events as well as a cell cycle arrest induction. To establish to what extent cell cycle arrest contributes to 1A-116 antiproliferative activity in GBM cells, we evaluated cell cycle progression by flow cytometry, synchronizing cells by starvation and stimulating them with FCS pulse. As seen in Figure 2G, 1A-116 arrested LN229 and T-98G cells in G0/G1 phase. Interestingly, 1A-116 50 μM totally blocked cell cycle progression compared to synchronized cells pulsed with FCS. In summary, 1A-116 is able to inhibit GBM cell proliferation in 2D and 3D settings by, at least in part, arresting cell cycle progression.

### 3.3. 1A-116 Is Predicted to Penetrate the Blood–Brain Barrier and Present a Favorable Metabolic Fate

Apart from efficacy and toxicity, pharmacokinetics and bioavailability are key features in drug development, being particularly important in intracranial tumor treatment. To start with, in these studies, we used different in silico approaches to predict 1A-116 metabolism and fate.

First, we determined gastrointestinal absorption and brain access since these two pharmacokinetic behaviors are crucial for considering 1A-116 as a feasible glioblastoma oral treatment. We used the predictive “Brain Or IntestinaL EstimateD permeation method (BOILED-Egg)” model [20], showing that 1A-116 is predicted to exert high gastrointestinal absorption and high blood–brain barrier permeability.

Finally, we evaluated whether 1A-116 is predicted to be a P-glycoprotein (P-gp) substrate. P-gp greatly influences the general pharmacokinetic parameters of clinically important therapeutics. In line with this, it has been shown that the expression of P-gp in cancer cells is a major cause of resistance to chemotherapy [26]. For this purpose, according to the SwissADME bioinformatic tool, 1A-116 is not a P-gp substrate. In line with this result, in another analysis made using the Biozyne software package, 1A-116 appears to be a poor P-gp substrate.

**Figure 3:**
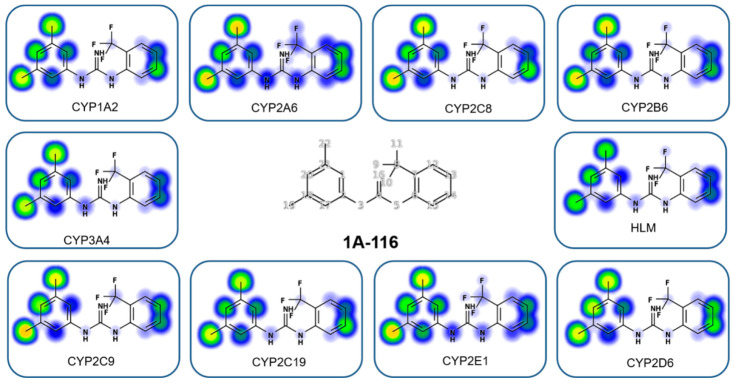
Predicted potential sites of metabolism (SOM) for 1A-116 using XenoSite predictor. Scores from 1 (red) to score zero (white) are shown on the 1A-116 chemical structure. Potential sites for nine CYP450 isoenzymes and HLM are shown.

Using the web-based Xenosite tool [22], it was possible to predict potential sites of metabolism (SOM) for 1A-116 for nine CYP450 isoenzymes and HLM. Results obtained from the in silico study are shown in Figure 3. Scores from 1 (red) to score zero (white) are depicted on the molecule. Interestingly, the output from XenoSite models can be interpreted as a probability of P450 metabolism. In the 1A-116 structure, there are predicted to be mainly two possible metabolic sites: C19 and C22. However, these two SOMs show intermediate scores, suggesting that the 1A-116 molecule would not be metabolized easily by the P450 complex of enzymes.

### 3.4. 1A-116 Shows a Favorable Toxicity Profile

In a non-clinical safety study, treated and control groups were examined daily for 14 days for general appearance, behavior, signs of toxicity, morbidity, and mortality. No mortality was observed during the study period. All the animals in the treated and control groups remained active and healthy during the experiment period. Therefore, as shown in Table 1 1A-116 treated groups showed no significant (*p* > 0.05) changes in either hematological or serum chemistry parameters. No macroscopic changes or structural abnormalities were observed in necropsy and histopathological studies (Figure S2. Supplemental Materials). The Maximum Tolerable Dose (MTD) was around 31.2 mg/kg, showing that 1A-116 ip administration is well tolerated up to that dose. Reversible toxic effects observed at 68 mg/kg ip treatment included trembling, ataxia, and heavy neurological symptoms. These results show that 1A-116 is well tolerated in mice and paves the way to establish effective doses in xenografts models.

Acute toxicology studies showed a LD50 of 98.11 mg/kg (95% confidence interval of 55 to 175 mg/kg) to ip administration and 2000 mg/kg to oral administration (95% confidence interval of 665.7 to 4540 mg/kg).

### 3.5. 1A-116 Elicits In Vivo Antitumor Activity in an Orthotopic Human Glioblastoma IDH-Wildtype Model

**Figure 4:**
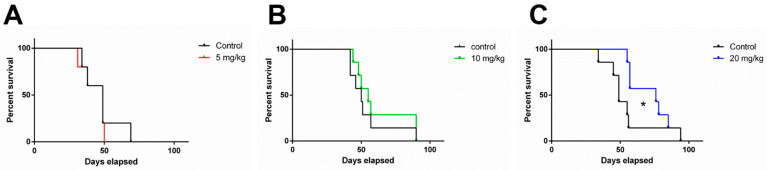
Glioma-bearing nude mice treated with different i.p daily doses of 1A-116. Survival curves of nude mice treated with vehicle or 1A-116 are shown. (**A**) 5 mg/kg/day 1A-116 daily treatment did not show differences compared to control group (n = 5 in each group). (**B**) 10 mg/kg/day 1A-116 treatment show a non-significant increase in median survival (n = 7 in each group). A representative experiment of two independent experiments is shown. (**C**) 20 mg/kg/day 1A-116 significantly increased mice survival compared to control group (n = 7, Mantel–Cox log-rank Test, * *p* < 0.05). A representative experiment of two independent experiments is shown.

Since 1A-116 showed antitumor effects on LN229 3D in vitro growth, the antitumor in vivo activity of 1A-116 was evaluated by using LN229 tumors grown as orthotopic xenografts in nude mice. To determine which Central Nervous System (CNS) tumor subtype belongs to this tumor cell line, we genotyped LN229 cells by MLPA since it could point out future clinical applications (Table 2). As shown in Figure 4A, LN229 is a wt IDH glioblastoma cell line showing a non-methylated MGMT promoter and wt TP53. EGFR, CDK4, and MIR26A2 are amplified.

LN229 cells were orthotopically implanted in mice striatum, and three different doses were evaluated in a 5-day/week schedule with ip administration.A total of 5 mg/kg/day showed no therapeutic benefit with no changes on median survival (Control: 49 days vs. 5 mg/kg/day: 49 days), while doses of 10 mg/kg/day increased mice median survival by 10% (Control: 50 days vs. 10 mg/kg/day: 55 days) and 20 mg/kg/day reached a significant 55% survival increase compared to the control group (Control: 49 days vs. 20 mg/kg/day: 76 days) (Figure 4B,C). These results show a dose-dependent therapeutic effect compared with vehicle-treated mice.

## 4. Discussion

The limited responses of gliomas to the standard of care (surgery, chemotherapy with TMZ, and radiotherapy) and high morbidity and mortality shown in this disease pose an urgent unmet medical need that necessitates the development of new targeted treatment options. To tackle the challenge of predicting the clinical efficacy of novel therapeutic agents targeting new molecular targets, it is imperative to seek robust target validation and establish relevant in vitro systems and clinically significant in vivo models for drug testing. Indeed, we provide evidence that Rac1 inhibition by the 1A-116 molecule may be a feasible strategy to treat gliomas.

To begin evaluating the possible impact of 1A-116 treatment in glioblastoma, we first analyzed Rac1 mRNA expression levels and its prognostic role in patient survival and outcome using publically available datasets. We found that Rac1 mRNA levels are upregulated compared to normal tissue, correlate with tumor grade, and higher expression levels are associated with a poor survival expectancy, as seen with other solid tumors [27]. Interestingly, there is significantly lower survival for those patients receiving TMZ and radiotherapy treatment with high levels of Rac1 expression. This could indicate that Rac1 is a key protein in the lack of treatment responsiveness and/or provides compensatory resistance mechanisms to standard therapy such as chemo- and radiotherapy, as described in other tumor types [4].

Even though two-dimensional (2D) cell culture is a simple and amenable system for rapid drug testing, it does not recapitulate the essential cellular organization and interactions that occur in vivo. Three-dimensional cell culture techniques often bridge the gap between 2D cultures and in vivo models [28]. Importantly, Eichler et al. compared both culture methods and used several measurement techniques showing that each glioma cell line, culture model, and therapeutic agent have no predictable responses, and 3D tumor models cannot be predicted by upscaling IC50 values obtained in 2D models [29]. In this work, we used scaffold-free 3D multicellular tumor spheroids to evaluate 1A-116 activity in vitro. These spheroids mimic metabolic and proliferation gradients of the in vivo tumor and are a clinically relevant model of chemotherapy resistance [30]. In line with this, here we show significant differences in 1A-116 sensitivity between 3D and 2D culture systems. Of interest, the proliferation of LN229 spheroids is dramatically reduced with 1A-116 concentrations over 25 μM, while in U87MG spheroids, this effect is shown in a concentration over 100 μM. Interestingly, Alfonso et al. thoroughly discussed the migration–proliferation dichotomy (also known as “Go-or-Grow” mechanism) of gliomas [31], where highly migratory cells have a lower proliferation rate compared to actively proliferating cells that move slowly. This seems to be the case of LN229 and U87MG cells, since control spheroids of U87MG seem to proliferate at a higher rate than LN229 spheroids. This idea is reinforced by a recent study demonstrating that LN229 and U87MG grow and infiltrate distinctively in the brain of a mouse model; U87MG glioma cells grow with well-demarcated tumor margins while LN229 cells grow with diffusive margins and represent human GBM more closely than U87MG cells [32].

Given the marked differences between both models and that Rac1 is attractive for therapeutically targeting invasion dynamics in glioma, we further established an orthotopic intracranial LN229 mouse model to evaluate 1A-116 efficacy. Our findings show a dose-dependent increase in survival of tumor-bearing mice in response to 1A-116 i.p treatment, with the 20 mg/kg/day treatment scheme showing significant antitumor efficacy. This first proof of concept calls the attention of two important venues to explore: combination schemes with TMZ and radiotherapy and an oral administration route for translating this therapy into a daily treatment for possible clinical applications. Intraperitoneal administration avoids the intestinal barrier since the molecule can move directly into systemic circulation through the peritoneal cavity. The oral route presents an additional challenge for systemic absorption through the gastrointestinal tract [33] but is key to patient adhesion to daily treatment. Our in silico predictions already show that an oral administration of 1A-116 is feasible since it is predicted to be absorbed gastrointestinally, and 1A-116 appears to be a poor substrate for P450 enzymes. Of interest, we also address by in silico predictions 1A-116 BBB penetration capacity. The existing “translational gap” in clinical trials for glioma has already been discussed [34], where BBB access and chemoresistance of glioma-stem-like cells are seldom addressed. Our work has already considered these key issues in drug development for glioma treatment. We next need to confirm these predictions using in vivo models, including a detailed pharmacokinetic study to establish 1A-116 plasma concentrations and brain bioavailability.

Finally, to define the translational significance of these results, it was important to establish the molecular signature of the LN229 model where 1A-116 showed antitumor efficacy. Central nervous system tumor classification has recently been updated by the World Health Organization [35], and molecular markers have gained a pivotal role in brain tumor taxonomy, and this could pave the way to precision medicine for patients. As shown here, the glioma in vivo model used in this study is IDH-wt, which is expected to respond to the TMZ treatment since the MGMT promoter is highly methylated. This kind of tumor type is observed in individuals over 60 years old as a de novo tumor. These results are in line with cell line characteristics shown in the ATCC repository because LN229 was isolated in 1979 from a 60-year-old female patient [36]. Future studies exploring other glioma types are important to establish which tumor types could be treated with this therapeutic approach.

## 5. Conclusions

Our emerging results suggest that the inhibition of Rac1 by 1A-116 may result in potential therapeutic benefits in glioma. Rac1 has long been recognized as an interesting molecular target in several solid and hematological tumors. Rac1 is involved in stemness maintenance, tumor cell invasion, and chemoresistance, all key features in glioma behavior and disease progression. However, Rho-GTPase targeted agents are still being researched, with several studies aiming to understand and predict their clinical value. Here we report 1A-116, a Rac1 inhibitor with a favorable toxicological profile that proves to present in vivo efficacy. Even though we are showing 1A-116 activity as a single agent in this first report, there is an ever-increasing interest in the combination of several therapeutic agents to tackle this incurable disease that still needs to be addressed. Altogether, our study establishes a strong potential for clinical translation of 1A-116 as a signal transduction-based precision therapy for glioma, as well as increases the evidence of Rac1 as a key molecular target.

## Figures and Tables

**Table 1:** Hematology and serum chemistry parameters of mice treated with 1A-116 and controls.

Parameter	Control Animals (n = 5)	Treated Animals	
		31.2 mg/kg	68 mg/kg
**HCT (%)**	46.51 ± 4.47	42.2 ± 0.83	40.0 ± 2.44
**RBC (×106/µL)**	9.84 ± 0.72	9.46 ± 1.71	10.09 ± 0.61
**HGB (g/dL)**	12.02 ± 0.76	11.8 ± 0.26	13.47 ± 0.68
**WBC (×103/µL)**	6.106 ± 1.316	6.251 ± 1.310	7.030 ± 0.718
**NEU (×103/µL)**	1.864 ± 0.413	1.753 ± 0.0371	2.022 ± 0.256
**EOS (×103/µL)**	0.065 ± 0.039	0.081 ± 0.061	0.088 ± 0.038
**BAS (×103/µL)**	0	0	0
**LYM (×103/µL)**	3.967 ± 0.881	4.176 ± 0.806	4.711 ± 0.927
**MNO (×103/µL)**	0.208 ± 0.143	0.257 ± 0.224	0.208 ± 47.65
**ALT (UI/L)**	41.20 ± 5.67	42.00 ± 14.94	39.00 ± 5.35
**AST (UI/L)**	123.60 ± 14.22	131.00 ± 19.33	149.75 ± 16.05
**GGT (UI/L)**	5.20 ± 1.31	4.60 ± 0.54	6.00 ± 1.00
**AMY (UI/L)**	376.00 ± 56.46	389.8 ± 73.27	333.0 ± 14.98

RBC: red blood cells, HGB: hemoglobin, HCT: hematocrit, WBC: white blood cell, NEU: eutrophils, EOS: eosinophils, BAS: basophils, LYM: lymphocyte, MNO: monocyte, ALT: alanine aminotransferase, AST: aspartate aminotransferase, GGT: gamma glutamyltransferase, AMY: amylase. Data are presented as mean ± standard deviations. No statistically significant differences compared with the control group at *p* < 0.05 were observed (analysis of variance, Dunnett post hoc test).

**Table 2:** LN229 genotyping using MLPA. Molecular markers with clinical importance were analyzed.

	LN229
**Mutations in IDH1 and IDH2**	
variant R132H—IDH1	Not detectable
variant R132C—IDH1	Not detectable
variant R172K—IDH2	Not detectable
variant R172M—IDH2	Not detectable
**Methylation status of MGMT promoter**	
	High (r = 099)
**Gene Amplification/Deletion**	
PDGFRA	Normal
EGFR	Ampl (r = 1.5)
EGFR vIII presence	Not Detectable
CDKN2A	Del (r = 0.0)
PTEN	Normal
CDK4	Ampl (r = 1.47)
MIR26A2	Ampl (r = 1.38)
MDM2	Normal
NFKBIA	Normal
TP53	Normal

IDH: Isocitrate dehydrogenase; PDGFRA: platelet-derived growth factor receptor alpha; EGFR: epidermal growth factor receptor; CDKN2A: cyclin-dependent kinase inhibitor 2A; PTEN: Phosphatase and tensin homolog; CDK4: Cyclin Dependent Kinase 4; MIR26A2: MicroRNA 26a-2; MDM2: MDM2 proto-oncogen; NFKBIA: Nuclear Factor Kappa B Subunit inhibitor A; TP53: Tumor Protein P53.

## Data Availability

The data presented in this study are available in this article (and Supplemental Materials).

## Supplementary Materials

The following supporting information can be downloaded at: https://www.mdpi.com/article/10.3390/cancers14194810/s1. Figure S1: Representative histological images of brain from mice injected with LN229 cells on day 6 comparing to mock mice. (A) Mock animal, (B) and (C) LN229- tumor bearing mouse brain. (Original magnification (A) and (B): 100×; (C): 400×). Figure S2: Representative histological images of the (A) liver, (B) kidney, (C) adrenal gland, (D) lung, (E) heart and (F) brain from mice treated with of a dose of 68 mg/kg of 1A116 and sacrificed at day 14 day, showed that no significant lesions occurred. (Original Magnification: 400×).
