# Supplementary material for: Preclinical Efficacy and Toxicology Evaluation of RAC1 Inhibitor 1A-116 in Human Glioblastoma Models
Source: Cancers (Basel). 2022 Sep 30;14(19):4810. doi: 10.3390/cancers14194810 (PMC9562863; doi:10.3390/cancers14194810)
Supplement: Supplementary file 1 [file cancers-14-04810-s001.zip › cancers-1948111-supplementary.pdf]

Materials and Methods

Histopathological analysis

For toxicological studies, samples of liver, kidney, adrenal, lung, heart and brain were fixed in 4% buffered formaldehyde for 8-10 h at 25ºC and then washed in phosphate buffered saline (PBS). Then, fixed tissues were dehydrated in an ascending series of ethanol, cleared in xylene and embedded in paraffin. Sections (5 µm thick) were mounted on slides previously treated with 2% (v/v) 3-aminopropyltriethoxysilane in acetone (Sigma-Aldrich, St. Louis, MO, USA) and stained with haematoxylin-eosin. Microscopic images were digitized with a CCD colour video camera Nikon DS-Fi2 mounted on a conventional light microscope Nikon Eclipse Ci-L Ni (Tokyo, Japan) and were examined by a veterinary pathologist.

To confirm tumor presence in brain orthotopic model, on day 6 after surgery mice were euthanized and brains were fixed and processed as previously described. Microscopic images were observed and analysed using an inverted light microscope (Leica).

Results

Histopathological analysis of tumor presence

Histological examination was carried out to confirm LN229 tumor establishment on day 6 before starting 1A-116 treatment. As seen in Figure S1, presence of tumor cells were confirmed in H&E stained tissue in animals injected with LN229 cells.

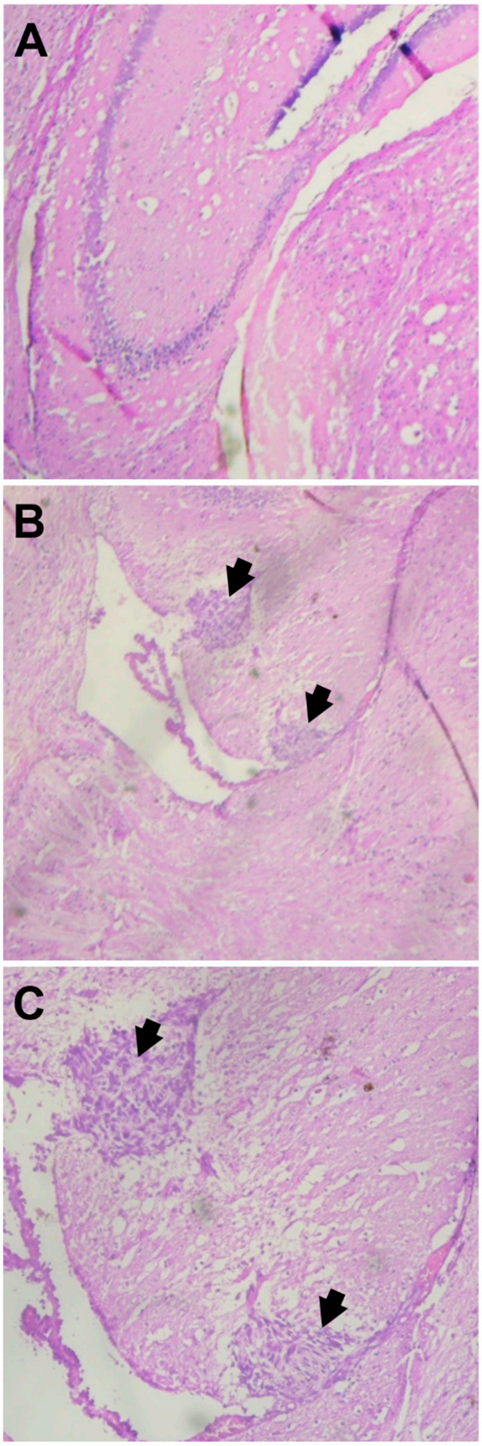

<u>Figure S1:</u> Representative histological images of brain from mice injected with LN229 cells on day 6 comparing to mock mice. (A) Mock animal, (B) and (C) LN229- tumor bearing mouse brain. (Original magnification (A) and (B): 100X; (C): 400X).

Histopathological analysis in toxicological studies

There were no treatment-related effects on histology and all the organs studied were normal without microscopic lesions (Figure S2).

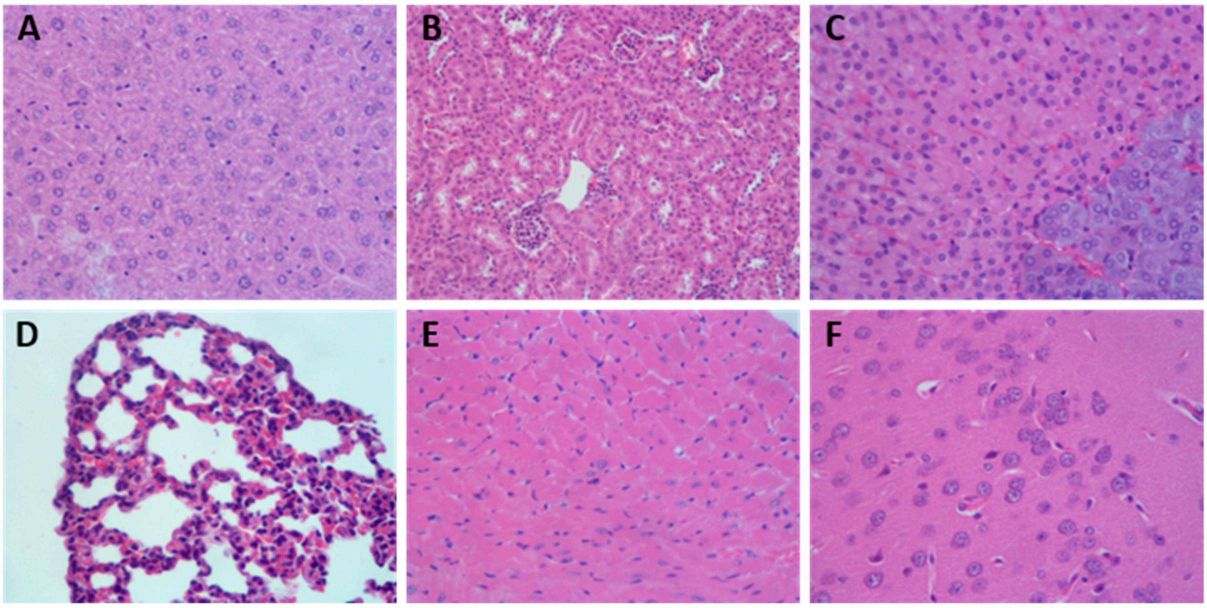

Figure S2: Representative histological images of the (A) liver, (B) kidney, (C) adrenal gland, (D) lung, (E) heart and (F) brain from mice treated with of a dose of 68 mg/kg of 1A116 and sacrificed at day 14 day, showed that no significant lesions occurred. (Original Magnification: 400X)
